# Menthol-Based Cream as a Novel Therapy for Diabetic Skin Wounds

**DOI:** 10.3390/pharmaceutics18010125

**Published:** 2026-01-19

**Authors:** Ana Júlia Vieira, Fernando Pereira Beserra, Gabriel Bacil Prata, Emanuel Ricardo Monteiro Martinez, Rafael Henrique Nóbrega, Luis Fernando Barbisan, Claudia Helena Pellizzon, Ariane Leite Rozza

**Affiliations:** Department of Structural and Functional Biology, Institute of Biosciences, São Paulo State University (UNESP), Dr. Antonio Celso W Zanin Street, 250, Botucatu 18618-689, Brazil

**Keywords:** menthol, diabetes mellitus, anti-inflammatory, skin, wound healing, antioxidant, IL-10, NO, natural products

## Abstract

**Background/Objectives**: Diabetes mellitus impairs skin wound healing by promoting a chronic inflammatory response and increased oxidative stress. This study aimed to investigate the healing potential of menthol in skin wounds of diabetic rats. **Methods**: A single dose of streptozotocin (50 mg/kg, i.p.) induced type 1 diabetes mellitus in male Wistar rats. After nine days, a skin wound was made on the rats’ back and treated with vehicle, insulin-based cream (0.5 U/g), or menthol-based cream (0.5%) for 14 days. After the euthanasia, the wound area was destined for assays of anti-inflammatory and antioxidant activity, protein expression levels by Western blotting, measurement of MPO activity, and quantitative mRNA expression. Nitrite levels were measured in blood plasma. **Results**: The group treated with menthol-based cream decreased the wound area by 94%. Also, menthol reduced the levels of TNF-α and IL-6 and increased IL-10 levels, besides stimulating the activity of antioxidant enzymes SOD, GPx, and GR, and enhancement in GSH and nitrite levels. Menthol downregulated the expression of *Nfκb* and upregulated the *Il10* and *Ki67* gene expression and the eNOS protein expression. **Conclusions**: Topically applied menthol accelerated the skin wound healing in diabetic rats through anti-inflammatory and antioxidant activities and increased cell proliferation, supporting its potential as a therapeutic strategy for diabetic wound management.

## 1. Introduction

Diabetes mellitus is a chronic metabolic disorder characterized by hyperglycemia caused by a deficiency in insulin secretion and action. In type 1 diabetes mellitus, the immune system acts against the pancreatic β-cells, causing the apoptosis of these cells responsible for the production and secretion of insulin. According to International Diabetes Federation, 589 million people worldwide are currently affected by diabetes [1].

Up to approximately 34% of individuals with diabetes will develop a skin wound during their lifetime, representing one of the most common factors contributing to hospital admission among patients with diabetes [2]. Each year, an estimated 18.6 million people with diabetes develop a foot ulcer [3]. Of these, up to 20% require hospitalization, and nearly one fifth of hospitalized patients subsequently undergo lower extremity amputation, primarily due to infection and progressive gangrene [4]. Worldwide, approximately 1.6 million amputations are performed annually as a consequence of infected diabetic wounds [3,5]. In the last 20 years, the costs of treating diabetes-related chronic wounds have extensively increased, and it is about 3.2 billion dollars in the United States, considered a public health problem [6].

Impaired wound healing in individuals with diabetes is driven by a constellation of pathophysiological disturbances including chronic hyperglycemia-induced microvascular dysfunction, persistent inflammation, oxidative stress, neuropathy, and compromised angiogenesis, all of which interfere with normal cellular responses and tissue repair mechanisms [7,8,9]. These factors prolong the inflammatory phase and reduce effective granulation and re-epithelialization, predisposing diabetic wounds to infection and chronicity [7,9]. Current management strategies on the market encompass advanced dressings, growth factor-enriched formulations, and biomaterial-based delivery systems, such as polymeric or metallic nanocarriers designed to enhance localized antioxidant, antibacterial, and pro-angiogenic activity, with several phytochemical-based and nanoparticle-integrated products under investigation to improve healing outcomes in chronic diabetic wounds [7,10].

The skin wound healing process has three overlapping phases: inflammatory, proliferative, and remodeling of extracellular matrix. Under diabetic conditions, the inflammatory phase is late and prolonged due to increased neutrophils and macrophages migration at the wound area. These cells produce pro-inflammatory cytokines and reactive oxygen species [11]. At the proliferative phase, fibroblasts rise, which start to close the wound and are responsible for collagen deposition [12]. At the remodeling phase of the skin wound healing process, some fibroblasts develop into myofibroblasts, which exert a contraction that will result in the closure of the wound. These two phases are also impaired by diabetes, resulting in a late and irregular healing process [13].

In preclinical studies, an alternative to mimic type 1 diabetes is the use of drugs that promote the destruction of β-cells, leading to a lack of insulin production and, consequently, hyperglycemia. Streptozotocin has been successfully used to induce diabetes mellitus in studies of skin wound healing [14,15]. Streptozotocin enters pancreatic β-cells via GLUT2 receptors and causes alkylation of the genetic material, resulting in the formation of reactive oxygen species that contribute to the necrosis of the β-cells [16].

As diabetes mellitus is a worldwide health problem, researchers have been studying the use of natural products and compounds, in order to find new treatments for diabetic wounds [17,18,19]. Menthol, a cyclic monoterpene, is the primary compound found in the essential oil extracted from leaves of plants of the genus *Mentha*. The food, cosmetics, and pharmaceutical industries widely utilize this compound, one of the most flavoring substances [20]. Menthol has biological activities described in the literature, such as local anesthetic [21], analgesic [22], antibacterial [23], antifungal [24], and antiulcerogenic activity [25]. Our research group published a paper evidencing the wound healing effect of menthol in normoglycemic rats [26]. At this time, we aimed to study the skin wound healing effect of menthol in hyperglycemic rats, after the development of diabetes mellitus induced by streptozotocin, analyzing its effects on inflammation, oxidative stress, histological structure, and alterations in mRNA and proteins expression.

## 2. Materials and Methods

### 2.1. Experimental Animals

We used male Wistar rats weighing 250–300 g, acquired from the Central Animal House of UNESP in Botucatu. The rats were hosted in cages, with controlled temperature (21 ± 2 °C) and humidity (60 ± 1%) with 12 h light–dark cycles. Rats had free access to tap water and diet. All efforts were made to minimize animal suffering. The UNESP Institutional Animal Care and Use Committee (permit number 793-CEUA) approved the experimental protocols, which followed the Canadian Council on Animal Care recommendations.

### 2.2. Diabetes Induction

After the acclimatization period, the rats were submitted to the glycemic dosage to determine the normal glycemic levels. The glycemic parameters were defined by the enzymatic glucose oxidase method using blood samples collected from the animal’s tail, using Accu-Chek Active glucometer (Roche Diagnostics, São Paulo, Brazil). Then, the rats received a single dose of streptozotocin (50 mg/kg, Sigma-Aldrich, Saint Louis, MO, USA), diluted in citrate buffer solution (0.1 M, pH 4.5), intraperitoneal route [27]. After two days, rats with glycemic levels ≥ 250 mg/dL were considered diabetic.

### 2.3. Formulation of Creams

(−)-Menthol (catalog #63660, <99% purity) was purchased from Merck (Darmstadt, Germany) and incorporated into Lanette cream at 0.5% (*v*/*w*). Insulin cream was incorporated into Lanette cream and used as a reference drug (0.5 U/g). Lanette cream alone was used as the vehicle group.

### 2.4. Wound Creation and Treatment

One week after the diabetes establishment, all animals were conducted to wound procedure. At first, an intraperitoneal injection of ketamine (80 mg/kg) and xylazine (10 mg/kg) anesthetized the rats before a single subcutaneous injection of ketoprofen (100 mg/kg) to alleviate postoperative discomfort. We removed the hair from the rats’ backs and performed a skin wound using a 2 cm diameter punch. After the wounding procedure, the rats were moved to individual cages and randomly distributed into three groups (*n* = 10): vehicle, insulin cream (0.5 U/g), and menthol-based cream (0.5%). Once a day, the wound region was measured using a transparent plastic film, then photographed using a ruler as a scale. Furthermore, we applied the creams to skin wounds. We treated rats for 14 days. An increased depth of anesthesia euthanized the rats in the end. We collected the skin wound for biochemical and molecular assays, the liver for antioxidant assays, and the blood to perform nitric oxide measurement.

### 2.5. Wound Area Contraction

We calculated the wound area contraction using Adobe Photoshop CS3 (San José, CA, USA), using the following equation: [(wound area at day 1—wound area at day 14)/wound area at day 1] × 100. Values were expressed as the percentage of wound contraction [28].

### 2.6. Histological Analysis

Skin wound samples (*n* = 4 per group) were immersed in a fixative solution (85% alcohol 80, 10% formalin, and 5% acetic acid) and processed in a paraffin tissue processing machine. Sections of the skin wounds were cut to a thickness of 5 µm and stained with hematoxylin-eosin (HE) or Masson’s trichrome for histological evaluation. Photomicrographs were obtained using a camera (Leica DMC 2900, Wetzlar, Germany) attached to the microscope (Leica DM 2500).

### 2.7. Cytokines Level Measurement

Skin samples (*n* = 10 per group) were diluted in phosphate-buffered saline (1:10) and centrifuged at 4 °C. Cytokines were detected in the supernatant using commercial ELISA kits for TNF-α, IL-6, and IL-10 from R&D Systems (Minneapolis, MN, USA). All procedures followed the manufacturer’s protocols.

### 2.8. mRNA Expression by RT-qPCR Method

The total RNA of the skin wound (*n* = 10 per group) was extracted, using TRIzol^®^ reagent (Invitrogen, Carlsbad, CA, USA) and treated to avoid DNA contamination (DNase I, Invitrogen, Carlsbad, CA, USA), and then cDNA synthesis was performed, using Supercript^®^ II (Invitrogen, Carlsbad, CA, USA) [29]. Following the manufacturer’s instructions, the qPCR analysis was performed in duplicate in the StepOne system (Life Technologies, Carlsbad, CA, USA), and relative mRNA expression profiles were calculated according to Vischer et al. [30]. Cq values were performed using SYBR Green (Invitrogen, Carlsbad, CA, USA) based on the reference gene β-actin and then calibrated with ddCT; the vehicle group was used as the reference. qPCR was conducted using designed and specific primers for *Rattus norvegicus* (Table 1).

### 2.9. Antioxidant Assays

To prepare the liver homogenate (*n* = 10 per group), we used an extraction buffer (HEPES 50 mM pH 7.9, KCl 1 M, MgCl_2_ 1 M, NaCl 5 M, EDTA 0.1 M, and NaF 0.1 M) and protease cocktail inhibitor, and then all samples were centrifuged at 4 °C, 14,000 rpm for 45 min.

#### 2.9.1. Measurement of Glutathione (GSH) Levels

The liver homogenate was diluted in phosphate buffer (0.1 M, pH 7.4) and then added to the microplate containing TRIS (1 mM) and EDTA buffer (2 mM, pH 8.2). 5′5′-dithiobis-2-nitrobenzoic acid (DTNB) was added to complete the reaction. The yellow color was read at 412 nm on a spectrophotometer, and the result obtained was in nm of GSH in mg of protein [31].

#### 2.9.2. Glutathione Peroxidase (GPx) Activity

The liver homogenate was diluted in phosphate buffer (0.1 M, pH 7.4) and mixed with a cocktail containing GR, GSH, and NADPH, and hydrogen peroxide. The enzyme activity was determined by measuring the consumed NADPH per minute per mg and read at 365 nm every minute for ten minutes [32].

#### 2.9.3. Glutathione Reductase (GR) Activity

The GR activity was established according to Carlberg and Mannervik [33]. The assay was conducted using phosphate buffer (0.1 M, pH 7.4), EDTA (0.2 mM), oxidized glutathione (1 mM) and NADPH (0.1 mM). NADPH consumption was read at 340 nm every minute for ten minutes, and the result was shown as nmol per min per mg protein.

#### 2.9.4. Superoxide Dismutase (SOD) Activity

The SOD activity was evaluated in liver homogenate diluted in phosphate buffer (0.1 M, pH 7.4). The method consists of superoxide generation caused by a reduction in nitroblue tetrazolium using a hypoxanthine–xanthine oxidase system (Sigma-Aldrich, St. Louis, MO, USA). The reaction was read at 560 nm every minute for ten minutes, and the result obtained was in SOD unit per milligram of protein [34].

### 2.10. Myeloperoxidase (MPO) Activity

In order to assess the neutrophils accumulation, MPO activity was measured in liver samples. The method consisted in mixing the supernatant with phosphate buffer (0.05 M, pH 6.8), O-dianisidine dihydrochloride, 0.0005% hydrogen peroxide, and MPO (5 U/mL), read at 460 nm every minute for ten minutes. The results were expressed in units of MPO in a gram of protein.

### 2.11. Protein Expression by Western Blotting

The skin samples (*n* = 10 per group) were homogenized using RIPA buffer (Merck, Darmstadt, Germany), centrifuged, and the supernatant was collected to quantify the protein levels, using the Bradford method [35]. About seven µg of skin homogenates were heated in Laemmli sample buffer (Bio-Rad, Milan, Italy) and electrophoresed on a 10% sodium dodecyl sulfate–polyacrylamide gel, then transferred to nitrocellulose membranes (Bio-Rad, Milan, Italy) and blocked with bovine serum albumin (BSA). The membranes were incubated overnight with anti-AKT (1:1000), anti-eNOS (1:1000), anti-ERK 1/2 (1:1000), and anti-β-actin (1:1000). All antibodies were acquired from Cell Signaling Technology (Danvers, MA, USA). After five steps of washing with TBS-T, the membranes were incubated with a secondary antibody for two hours. We used Clarity Max Western ECL Substrate (Bio-Rad, Milan, Italy) to visualize the band intensities. The densitometric analysis was performed using Image J software, version 1.45 (Bethesda, MD, USA).

### 2.12. Measurement of Nitrite

A spectrophotometric method based on the Griess reaction determined the nitrite levels in blood plasma (*n* = 10 per group) [36]. A spectrophotometer read the colorimetric reaction at 557 nm. The data was expressed as μmol of nitrite per μg of total protein.

### 2.13. Statistical Analyses

The results were analyzed using ANOVA, followed by Dunnett’s test or paired *t*-test, when applicable, and compared with the vehicle group. The results were presented as mean ± s.d. All analyses were performed using Graph Pad Prism, version 9 (Graph Pad Software, La Jolla, CA, USA). A value of *p* < 0.05 was considered significant.

## 3. Results

### 3.1. Glycemic Levels

**Figure 1:**
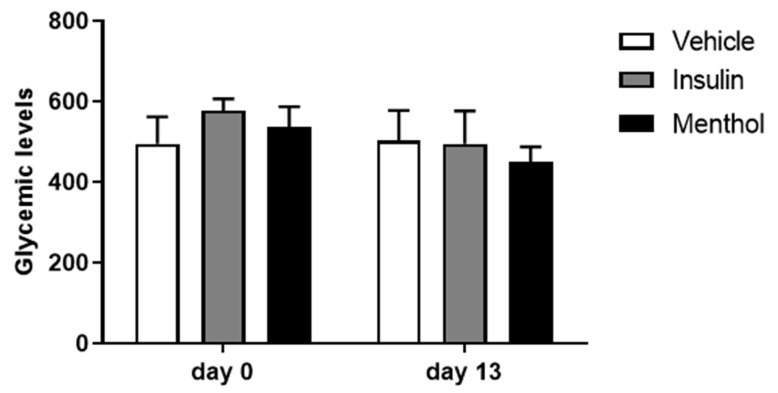
Glycemic levels (mg/dL) measured on the day before the wound (day 0) and a day before the euthanasia (day 13). Results are expressed as mean ± s.d. and analyzed by paired *t*-test.

The glycemic levels were measured the day before the wounding procedure and the day before the euthanasia. The results showed that the three groups maintained their glycemic levels above 400 mg of glucose/dL of blood, validating the establishment of diabetes in rats (Figure 1). The topical treatment in all groups did not alter the glycemic levels.

### 3.2. Wound Area Contraction

**Figure 2:**
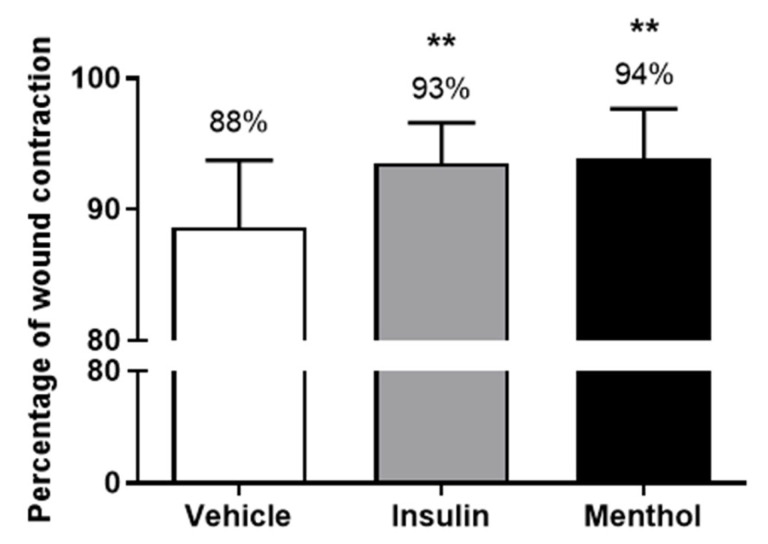
Percentage of skin wound contraction on the back of diabetic Wistar rats after 14 days of treatment with vehicle, insulin (0.5 U/g) cream, or menthol-based cream (0.5%). Results are expressed as mean ± s.d. and analyzed by ANOVA, followed by Dunnett, ** *p* < 0.01.

**Figure 3:**
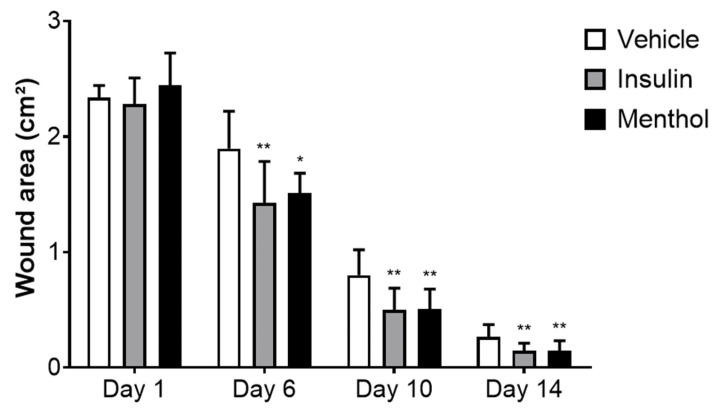
Wound area on the back of diabetic Wistar rats at day 1, 6, 10, and 14 of treatment with vehicle, insulin (0.5 U/g) cream, or menthol-based cream (0.5%). Results are expressed as mean ± s.d. and analyzed by ANOVA, followed by Dunnett, * *p* < 0.05, ** *p* < 0.01.

**Figure 4:**
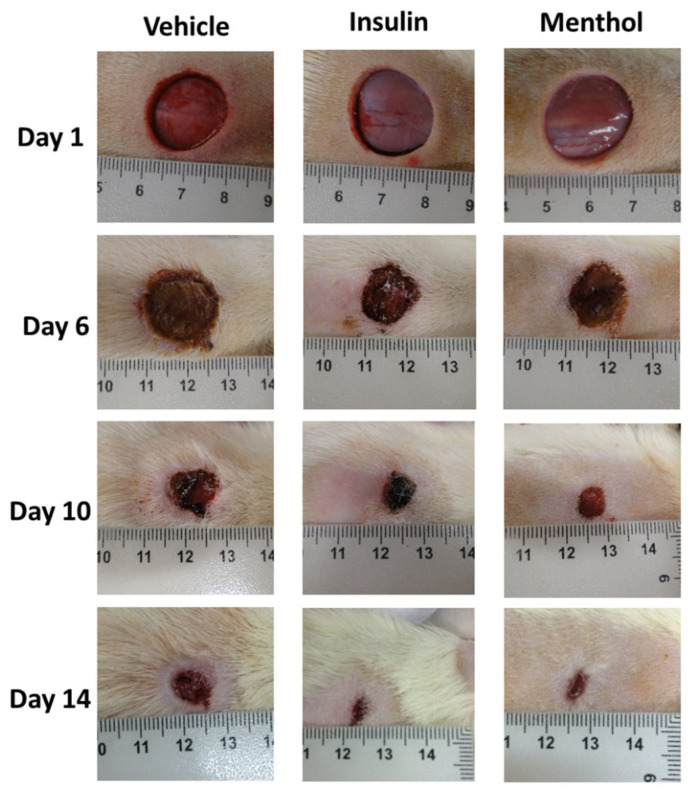
Representative images of skin wounds on the back of diabetic Wistar rats at day 1, 6, 10, and 14 of treatment with vehicle, insulin (0.5 U/g) cream, or menthol-based cream (0.5%).

The group treated with vehicle reduced the wound area by 88%, and the insulin cream-treated group by 93% after 14 days of treatment. The menthol-based cream presented the highest percentage of skin wound contraction (94%, *p* < 0.01, Figure 2). The skin wound healing progression on days 1, 6, 10, and 14 are shown in Figure 3 and Figure 4. From the sixth day of treatment, the groups treated with insulin cream and menthol-based cream showed a decrease in the wound area compared to the vehicle group. This reduction remained until the last day of treatment.

### 3.3. Histological Analyses

**Figure 5:**
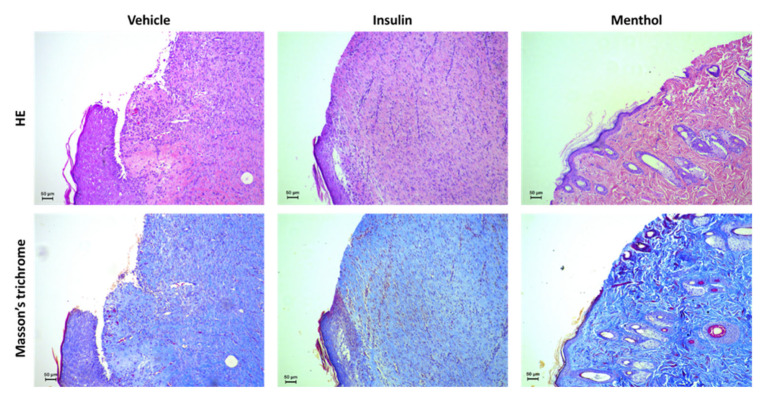
HE-stained and Masson’s trichrome-stained histological skin wound sections after 14 days of treatment with vehicle, insulin (0.5 U/g) cream, or menthol-based cream (0.5%).

The HE-stained slides showed that, after 14 days of treatment, the group treated with the vehicle still had the epidermal region undergoing regeneration compared to the groups treated with insulin cream and menthol-based cream, which showed an advanced repair process observed through an established epidermal layer and an organized extracellular matrix. The sections stained with Masson’s trichrome evidenced the organization of collagen fibers in the dermis of the rats treated with insulin cream and menthol-based cream (Figure 5). Masson’s trichrome is used in histological procedures to stain collagen fibers in blue [37].

### 3.4. Cytokines Level Measurement

**Figure 6:**
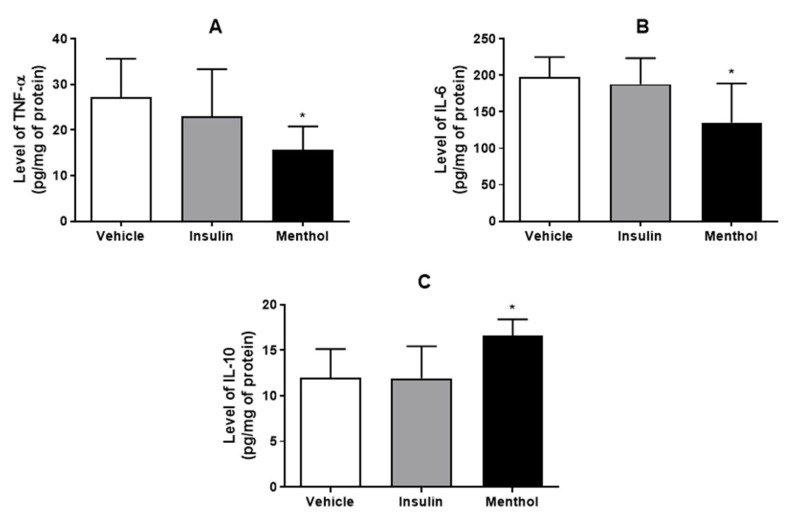
Levels of TNF-α (**A**), IL-6 (**B**), and IL-10 (**C**) in skin wound samples of diabetic Wistar rats after 14 days of treatment with vehicle, insulin (0.5 U/g) cream, or menthol-based cream (0.5%). Results are expressed as mean ± s.d. and analyzed by ANOVA, followed by Dunnett, * *p* < 0.05.

We measured the levels of pro-inflammatory cytokines TNF-α and IL-6, and anti-inflammatory cytokine IL-10, in skin wound samples. Under diabetic conditions, the pro-inflammatory cytokines are increased [38]. As shown in Figure 6, the levels of the cytokines were similar in the vehicle and insulin cream groups. In the menthol-treated group, the levels of pro-inflammatory cytokines decreased (*p* < 0.05), compared to the vehicle. The levels of IL-10, which inhibits the production of TNF-α and IL-6, were increased (*p* < 0.05).

### 3.5. mRNA Expression by RT-qPCR Method

**Figure 7:**
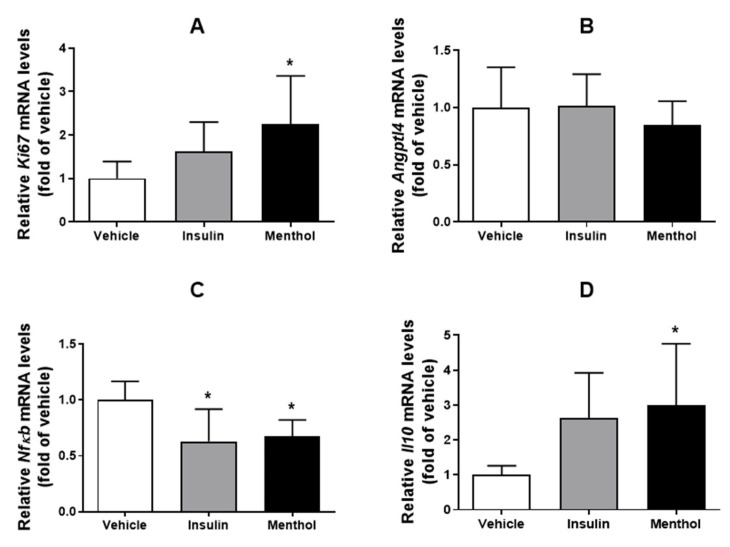
Quantity of *Ki67* (**A**), *Angptl4* (**B**), *Nfκb* (**C**), and *Il10* (**D**) mRNA expression, normalized with β-actin, in skin wounds of diabetic Wistar rats after 14 days of treatment with vehicle, insulin (0.5 U/g) cream, or menthol-based cream (0.5%). Results are expressed as mean ± s.d. and analyzed by ANOVA, followed by Dunnett test, * *p* < 0.05.

We quantified and analyzed the mRNA expression of *Ki67*, a proliferative cell marker, *Angptl4*, which plays a role in re-epithelization, *Nfκb*, a transcriptional factor responsible for regulating the inflammatory response, and *Il10*, an anti-inflammatory cytokine. As a result, as shown in Figure 7, the group treated with menthol-based cream showed increased *Ki67* and *Il10* mRNA expression (*p* < 0.05), besides decreased *Nfκb* mRNA expression (*p* < 0.05). There was no alteration in the *Angptl4* mRNA expression. The group treated with insulin cream presented levels of *Ki67*, *Angptl4*, and *Il10* mRNA expression similar to those of the vehicle group; however, it showed a significant reduction in *Nfκb* mRNA expression (*p* < 0.05).

### 3.6. Assays of Oxidative Stress

**Figure 8:**
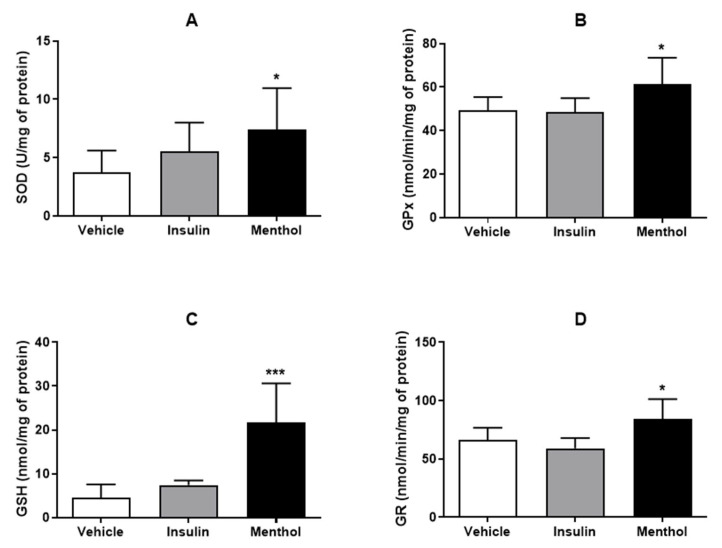
Levels of GSH (**A**) and enzymatic activity of GPx (**B**), GR (**C**), and SOD (**D**) in skin wounds of diabetic Wistar rats after 14 days of treatment with vehicle, insulin (0.5 U/g) cream, or menthol-based cream (0.5%). Results are expressed as mean ± s.d. and analyzed by ANOVA, followed by Dunnett, * *p* < 0.05, *** *p* < 0.001.

We measured the level of GSH and the activity of GPx, GR, and SOD enzymes. We observed that the group treated with menthol-based cream presented an enhanced level of GSH (*p* < 0.001) and high enzymatic activity of GPx, GR, and SOD (*p* < 0.05) in comparison to the vehicle, evidencing an antioxidant effect offered by menthol-based cream. Treatment with insulin cream did not alter GSH levels or the activities of antioxidant enzymes (Figure 8).

### 3.7. MPO Activity

**Figure 9:**
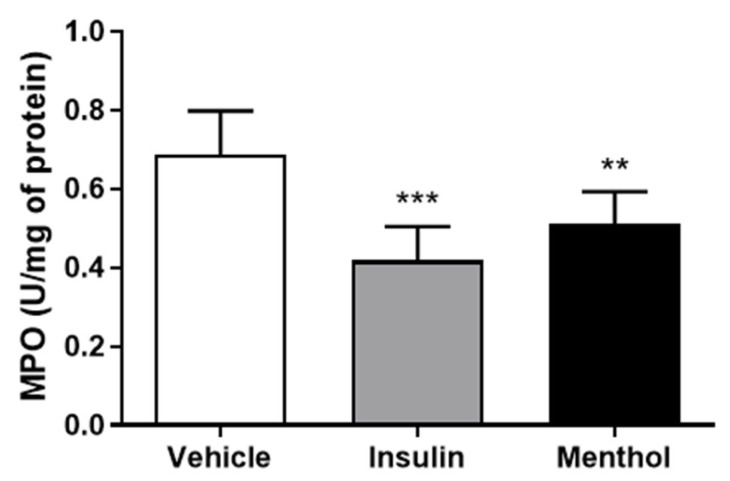
MPO activity in skin wounds of diabetic Wistar rats after 14 days of treatment with vehicle, insulin (0.5 U/g) cream, or menthol-based cream (0.5%). Results are expressed as mean ± s.d. and analyzed by ANOVA, followed by Dunnett, ** *p* < 0.01, *** *p* < 0.001.

Once infiltrated in the wound area, neutrophils produce MPO, which is well-known for being a pro-inflammatory marker and a pro-oxidant enzyme [39]. The activity of MPO decreased in the groups treated with insulin cream (*p* < 0.001) and the menthol-based cream (*p* < 0.01) groups (Figure 9).

### 3.8. Measurement of Nitrite Levels

**Figure 10:**
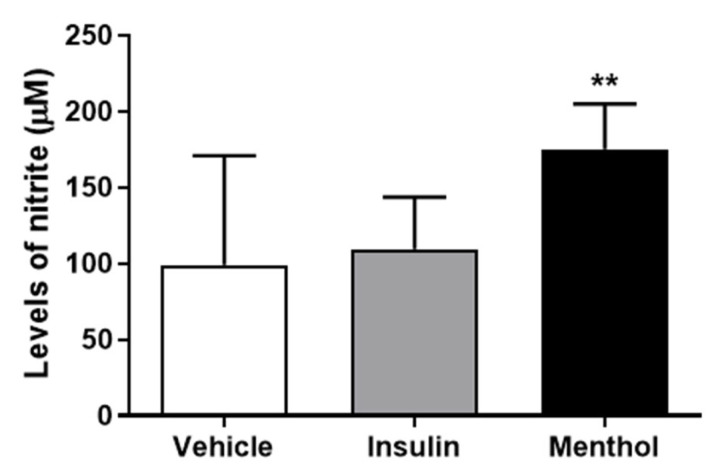
Nitrite levels (µM) measured in blood plasma after 14 days of treatment with vehicle, insulin (0.5 U/g) cream, or menthol-based cream (0.5%) in diabetic Wistar rats. Results are expressed as mean ± s.d. and analyzed by ANOVA, followed by Dunnett, ** *p* < 0.01.

NO is nitrite-dependent and plays a role in wound healing, modulating the re-epithelization, and collagen deposition [40]. After 14 days, the group treated with menthol-based cream showed an increase (*p* < 0.01) in nitrite levels in comparison to the vehicle group. Treatment with insulin cream resulted in nitrite levels similar to those of the vehicle group (Figure 10).

### 3.9. Protein Expression by Western Blotting

**Figure 11:**
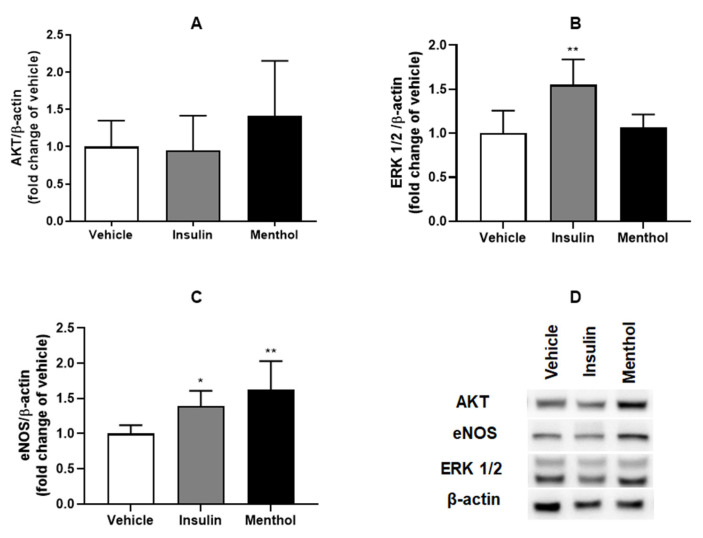
Relative protein expression of AKT (**A**), ERK 1/2 (**B**), and eNOS (**C**) and a representative image (**D**) of AKT, eNOS, ERK 1/2, and β-actin expression in skin wounds of diabetic Wistar rats after 14 days of treatment with vehicle, insulin (0.5 U/g) cream, or menthol-based cream (0.5%). Results are expressed as mean ± s.d. and analyzed by ANOVA, followed by Dunnett test, * *p* < 0.05, ** *p* < 0.01.

We analyzed the protein expression of AKT, ERK 1/2, and eNOS based on the expression of β-actin (Figure 11D). These proteins are related to cell growth, migration, and angiogenesis, which are involved in the wound healing process. Our results showed that the treatment with menthol-based cream did not interfere in the expressions of AKT (Figure 11A) and ERK 1/2 (Figure 11B). The relative expression of eNOS (Figure 11C) was increased in the groups treated with insulin cream (*p* < 0.5) and menthol-based cream (*p* < 0.01) in comparison to the vehicle. Treatment with insulin cream also increased the relative expression of ERK1/2 (*p* < 0.01, Figure 11B).

## 4. Discussion

The present study investigated the healing potential of a menthol-based cream in skin wounds on the back of streptozotocin-induced diabetic rats, as well as its underlying mechanisms of action. Wound healing under diabetic conditions is markedly impaired, mainly due to a persistent inflammatory response, excessive production of reactive oxygen species (ROS), increased susceptibility to infection, and microvascular damage [18]. These alterations compromise the orderly progression of the three healing phases, resulting in delayed and inefficient tissue repair. Our results demonstrated that topical treatment with the menthol-based cream accelerated wound healing in diabetic rats, with a significant reduction in wound area observed from the sixth day of treatment onward. This reduction was sustained until the end of the experimental period, indicating a consistent therapeutic effect. In contrast, wounds treated with vehicle alone exhibited a slower and incomplete healing process.

Histological analysis performed after 14 days of treatment further supported these findings. While the vehicle-treated group still showed features of an ongoing repair process, the menthol-treated group exhibited a well-established epidermal layer, the presence of newly formed blood vessels, hair follicles, and sebaceous glands, as well as an organized extracellular matrix with properly arranged collagen and elastic fibers, as evidenced by Masson’s trichrome staining. Taken together, the macroscopic and microscopic results indicate that menthol-based cream promoted not only faster wound closure but also a more effective and structurally organized tissue repair, characterized by the restoration of tissue integrity.

Inflammation plays a critical role in the early phase of wound healing; however, in diabetic conditions, this process is delayed and prolonged, impairing tissue regeneration [41]. NF-κB is a key transcription factor activated during wound healing, regulating genes associated with inflammation, oxidative stress, cell migration, proliferation, and adhesion. Under diabetic conditions, sustained NF-κB activation leads to excessive expression of pro-inflammatory cytokines, such as TNF-α and IL-6, which negatively affect healing [42]. Elevated TNF-α levels in diabetic wounds reduce cell proliferation and promote fibroblast apoptosis, while high IL-6 levels further delay the healing process [6]. In the present study, treatment with the menthol-based cream downregulated NF-κB expression, which was accompanied by decreased levels of TNF-α and IL-6. Additionally, both mRNA expression and protein levels of the anti-inflammatory cytokine IL-10 were increased. IL-10 is known to suppress the production of pro-inflammatory cytokines and to contribute to wound healing by modulating collagen deposition, preventing excessive scar formation, and promoting cell proliferation and tissue repair [43]. Although insulin cream has demonstrated a healing effect, our results indicate that it does not act through mechanisms that inhibit the inflammatory response. These findings indicate that menthol-based cream elicited a robust anti-inflammatory response that contributed significantly to the improvement of the wound healing process. Similar anti-inflammatory effects were previously observed in the skin wound of normoglycemic rats treated with menthol-based cream, which successfully exhibited increased IL-10 levels and reduced the expression of IL-1β, IL-6, and TNF-α after 14 days of treatment [26].

*Angptl4* expression was not altered among the experimental groups. Although previous studies have reported reduced *Angptl4* expression in diabetic skin wounds [44], the lack of modulation observed in the present study suggests that the beneficial effects of menthol-based cream on wound healing may occur independently of this pathway. The insulin cream also did not modulate *Angptl4* mRNA expression levels, as previously demonstrated by Beserra et al. [28].

Oxidative stress is another major factor contributing to impaired wound healing in diabetes. Chronic hyperglycemia increases MPO activity and ROS production, leading to oxidative damage that compromises angiogenesis, keratinocyte migration, and collagen deposition [18,45]. Endogenous antioxidant defenses, including enzymes such as SOD, GPx, and GR, as well as reduced GSH, play a crucial role in counteracting oxidative stress. In diabetic wounds, the activity of these enzymes and GSH levels are typically reduced, further aggravating tissue damage. Insulin cream, used as a positive control, did not exert its effect by stimulating antioxidant activity in the cells. The treatment with menthol-based cream significantly reduced MPO activity, indicating lower oxidative burden at the wound site. This reduction was associated with increased activities of SOD, GPx, and GR, as well as elevated GSH levels, suggesting an enhancement of the antioxidant defense system. These results indicate that menthol-based cream effectively attenuated oxidative stress, which likely contributed to the acceleration and improvement of the wound healing process.

The AKT/eNOS and ERK1/2 signaling pathways are involved in regulating cell growth, migration, and angiogenesis, primarily through the stimulation of NO production in endothelial cells [46]. NO has been widely explored as a therapeutic agent for non-healing wounds, including diabetic ulcers, due to its angiogenic and antimicrobial properties [47,48]. In this study, AKT expression did not differ among treatment groups; however, eNOS expression was significantly increased in the menthol-treated group. This increase likely explains the elevated nitrite levels observed, reflecting enhanced NO production. The increased NO availability may have contributed to the upregulation of *Ki67* mRNA expression, a marker of cell proliferation, thereby promoting tissue regeneration and angiogenesis. These findings suggest that menthol-based cream enhances wound healing in diabetic rats by modulating inflammatory and oxidative pathways and by stimulating NO-mediated cellular proliferation and vascularization.

## 5. Conclusions

This study demonstrates that a menthol-based topical formulation promotes effective skin wound healing under diabetic conditions by favorably modulating key biological processes involved in tissue repair. The coordinated regulation of inflammatory balance (decreased levels of TNF-α and IL-6 and increased IL-10 levels), oxidative stress (increased SOD, GR, and GPx activities, as well as GSH levels, together with decreased MPO activity), and pathways associated with angiogenesis and cell proliferation (increased nitrite levels, downregulation of *Nfκb* gene expression, upregulation of *Il10* and *Ki67* gene expression, and increased eNOS protein expression) suggests that menthol acts on multiple targets within the wound microenvironment. From a translational perspective, the topical delivery of menthol represents a promising strategy for diabetic wound care, offering localized therapeutic action with reduced systemic exposure. These findings provide a mechanistic rationale for further translational studies and support the continued development of menthol-based formulations as potential adjunctive therapies for impaired wound healing associated with diabetes.

## Figures and Tables

**Table 1:** Specifications of primers used in RT-qPCR.

Target	Size	Sequence 5′–3′	Melting Temperature	NCBI Reference Sequence
*Ki67*	100	F: GGGTTTCCAGACACCAGACC	60 °C	NM_001271366.1
		R: ACCAGGAAGACCAGTTAGAACC		
*Angptl4*	90	F: AACTGTTCCAGAAGGTAGCCC	60 °C	NM_199115.2
		R: TCAAGAGGTCAATCTGGCTCTG		
*Nfκb*	98	F: CCTCATCTTTCCCTCAGAGCC	60 °C	NM_199267.2
		R: CGCACTTGTAACGGAAACGC		
*Il10*	95	F: GACGCTGTCATCGATTTCTCC	60 °C	NM_012854.2
		R: GCTCCAAGACAAAGGTGTCTAC		
*β-actin*	80	F: CCCTGGCTCCTAGCACCAT	60 °C	NM_031144.3
		R: GATAGAGCCACCAATCCACACA		

F: forward, R: reverse.

## Data Availability

Data will be provided upon request.
